# Morphometric Analysis of the Clavicles in Chinese Population

**DOI:** 10.1155/2017/8149109

**Published:** 2017-04-12

**Authors:** Jesse Chieh-Szu Yang, Kun-Jhih Lin, Hung-Wen Wei, Cheng-Lun Tsai, Kang-Ping Lin, Pei-Yuan Lee

**Affiliations:** ^1^Division of Joint Reconstruction, Department of Orthopaedics, Taipei Veterans General Hospital, Taipei, Taiwan; ^2^Department of Electrical Engineering, Chung Yuan Christian University, Taoyuan, Taiwan; ^3^Technology Translation Center for Medical Device, Chung Yuan Christian University, Taoyuan, Taiwan; ^4^Department of Physical Therapy and Assistive Technology, National Yang-Ming University, Taipei, Taiwan; ^5^Department of Biomedical Engineering, Chung Yuan Christian University, Taoyuan, Taiwan; ^6^Department of Biomedical Engineering, National Cheng Kung University, Tainan, Taiwan; ^7^Department of Orthopedics Surgery, Show Chwan Memorial Hospital, Changhua, Taiwan

## Abstract

The clavicle has a complex geometry that makes plate fixation technically difficult.* The current study aims to measure the anatomical parameters of Chinese clavicles as reference for plate design*. One hundred clavicles were analyzed. The clavicle bone model was reconstructed by using computed tomography images. The length, diameters, and curvatures of the clavicle were then measured. The female clavicle was shorter, more slender, and less curved in lateral part than the male clavicle. There was a positive relationship between height and clavicle parameters except lateral curve and depth. The measurements of Chinese clavicles were generally smaller than Caucasians. The clavicle curves were correlated with the bone length; thus consideration of the curve variations may be necessary as designing size distribution of clavicle plate.

## 1. Introduction

The incidence of clavicle bone fracture among all fractures was 5~10%, and about 80% of clavicle bone fracture occurred in the midshaft of the bone [1, 2]. The interventions of midshaft clavicle fracture were controversy, with most fractures (89%) healed without operation involved [2–4]. However, recent study has proven that the prognosis of plate fixation in treating displaced midshaft clavicle fracture was better than nonoperative treatment [5, 6]. But still, the hardware-related problem remains to be the most common complication encountered after surgery [7, 8]. Recent literatures claimed that precontoured clavicular bone plate significantly decreases hardware prominence problems [8] and the precontoured plating system had lower rate of plate prominence versus noncontoured plating in patients who did not undergo hardware removal [9]. However, these cases were studied between Caucasian population and specific precontoured Locking Clavicle Plates (Acumed, Hillsboro, Oregon; Depuy-Synthes, PA, USA). It is well known that most of commercially available orthopaedic devices are designed according to the anthropometric data of* Caucasians*, which has been deduced as the cause of the component mismatch in Asian population [10–15]. Anatomy of the clavicle is quite complex due to its S-shaped curvature and cephalad-to-caudad bow. Several authors have measured the morphological parameters of the clavicle from the Western donors [16, 17]. To our knowledge, no anthropometric research exists evaluating the morphology of the clavicle for Asians.* This study aims to measure the geometric parameters of the CT-based clavicle model for Chinese population.* We used three-dimensional (3D) computational modeling to pursue quantitative understanding of the clavicle.

## 2. Materials and Methods

### 2.1. Reconstruction of Three-Dimensional Clavicle Model

The upper extremity computed tomography (CT) scans were completed and evaluated at Show Chwan Memorial Hospital and the study was approved by the Institutional Review Board, Show Chwan Memorial Hospital (SCMH IRB number 1021004). One hundred clavicle bones (50 male and 50 female) with average age of 37.0 (range 20–59 years) were collected. None of these bones had former pathologic problems or fractures. The CT scans (Light Speed VCT, GE Medical System, General Electric Company, USA) (protocol: voltage 120 kV, pitch 0.984, standard reconstruction kernel) were collected with* slice thickness of 0.625 mm and 512 × 512 pixels per image*. The subjects were scanned for CT in a supine position. The right clavicle was studied and CT scanned from proximal to distal end. Image-processing software* (Amira 4.1, Mercury System, MA, United States)* was used to draw the cortical outline for reconstructing the bone model.* Cortical bone contour was retrieved by adjusting the gray level threshold of the CT image. The STL files of the clavicle bone models were then exported to SolidWorks software (SolidWorks Corp., MA, USA).* SolidWorks was used to perform geometric measurements.

### 2.2. Measurement of the Morphological Parameters

**Figure 1:**
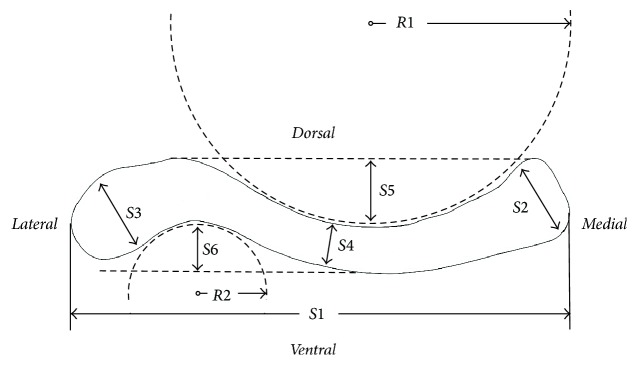
Clavicle geometrical measurements: general length (*S*1), sternal diameter (*S*2), acromial diameter (*S*3), middle diameter (*S*4), depth of the medial curve (*S*5), depth of the lateral curve (*S*6), medial curve radius (*R*1), and lateral curve radius (*R*2).

The morphological parameters of the clavicle bone including total length, the diameters of three different regions, and the curvatures of the arched segment were measured according to the research of Andermahr et al. [16], as shown in Figure 1.* The measurements include (1) the length of the clavicle (S*1*), distance between the proximal and distal ends of the clavicle, (2) the sternal diameter (S*2*), diameter of a best-fit circle fitted to the profile of the sternal end, (3) the acromial diameter (S*3*), diameter of a best-fit circle fitted to the profile of the acromial end, (4) the midshaft diameter (S*4*), diameter of a best-fit circle fitted to the profile of the middle shaft, (5) the depth of the medial curvature (S*5*), perpendicular distance between the deepest point of the medial concavity and a tangent that touched the lateral convexity and the dorsal bone surface of the medial part of the clavicle, (6) the depth of the lateral curvature (S*6*), measured similarly as S5, (7) the radius of the medial curvature (R*1*), radius of a best-fit circle fitted to the profile of the medial concavity, and (8) the radius of the lateral curvature (R*2*), radius of a best-fit circle fitted to the profile of the lateral concavity.* All measurements were studied in the craniocaudal view and carried out via the built-in measurement tools of SolidWorks.

### 2.3. Statistical Methods

Statistical analysis was performed using SPSS Statistics Base 20.0. *T*-test was used to compare differences of each clavicular parameter between males and females. The significance level was defined as *P* < 0.05. The relationship between the height and the anatomical parameters was analyzed by using regression analysis.

## 3. Results

The demographic data are shown in Table 1.* Males in our sample (mean height of 171.14 cm) were significantly taller than the females (mean height of 159.50 cm) (P* < 0.0001*). Each of the clavicle dimensions measured* is summarized in Table 2 for the total group and for comparison between males and females.  Table 3 shows the relationship between the height and each anatomical parameter.

### 3.1. Length and Diameters

The average length was 15.6 ± 0.9 in male and 14.3 ± 1.3 in female (Table 2), which was statistically different (*P* < 0.001),* meaning the male clavicle is longer than the female's*. The bone diameters, including sternal end, acromial end, and middle shaft in male group, were significantly greater than those of females. Among these three diameters, the midshaft diameter was smaller whereas it was similar for the sternal and acromial ends.

### 3.2. Curvatures

The radius of the medial curvature (7.2 ± 1.5 cm) was significantly larger than that of the lateral curvature (3.5 ± 1.7 cm) (*P* < 0.001). Similarly, the medial curve was significantly deeper than the lateral curve (*P* < 0.001). For gender comparison, the medial curve depth, the medial curve radius, and the lateral curve depth for males were significantly larger than those of the females (1.7 ± 0.3 cm versus 1.5 ± 0.3 cm; 7.5 ± 1.4 cm versus 6.8 ± 1.6 cm; and 1.3 ± 1.1 cm versus 1.0 ± 0.2 cm) (*P* < 0.001; *P* < 0.05; *P* < 0.05) (Table 2). However, no gender-specific difference was noted on the lateral curve radius (3.4 ± 1.7 cm versus 3.7 ± 1.8 cm). Although it was statistically nonsignificant, the females had slightly greater lateral curve radius than the males.

### 3.3. Relationship between the Height and the Clavicle Parameters

Table 3 shows the relationship between the height and the clavicle parameters. Pearson correlation coefficient demonstrated a strong positive correlation (*r* = 0.84) between the height and the clavicle length (*P* < 0.0001). There were also moderately strong positive correlations between the height and clavicle diameters and between the height and the medial curvature. In contrast, lateral curve depth and radius were found to be of weak correlation to height.

## 4. Discussion

Anatomical precontoured plates have been demonstrated as good alternatives for the treatment of clavicle fractures. With the precontoured shape, the clavicle plate can reduce hardware prominence, soft tissue irritation, and rate of reoperation for implant removal [8, 9]. However, commercially available precontoured clavicle plates were generally designed for Western populations [7] which may be improper for Eastern population. It is well known that bony morphology differences exist between different ethnic groups [10–15]. Several literatures have reported the measurement data of the clavicle from Caucasians [16, 17]. To the authors' knowledge, there are no studies in the literature characterizing anthropometric parameters of the Chinese clavicle as it relates to implant design. This study was meant to measure the dimensions of the clavicles of Chinese subjects and to compare these measurements with the dimensions of other ethnic group.

The average clavicle length for our sample was* 14.9* cm.* This value is slightly smaller than the values from Andermahr et al. (15.1 cm) [16] but slightly larger than those of Duprey et al. (14.7 cm) [17] (Table 4)*. More importantly,* most of* the clavicle diameters and the radii of curvatures measured in the present study were smaller than the values reported by Andermahr et al. [16] and Duprey et al. [17]* except S*4* and R*1. For a small bone diameter, it indicated that clavicles of our sample (Chinese) were more slender than other studies (Caucasians). In addition, the* depth* of the medial and lateral curvatures was smaller in our sample than in the Caucasians [16, 17], meaning that the shape of the clavicles for Chinese is more curved in craniocaudal view.* Nevertheless, the differences between the Chinese and the Caucasian measurements are mild*. Otherwise, in the clavicles parameters, it was noted that the radius of the medial curvature for males was greater than that of females. On contrary, females have greater lateral curve radius than males. This gender difference may require different plate designs for male and female patients with midshaft clavicular fractures. Andermahr et al. [16] also revealed identical finding with our study.

The clavicle specimens in the current study were obtained from 3D model reconstruction and in Andermahr et al.'s study [16], they were cadaveric bones. Although the sources were different, the measurements all were based upon a 2D viewing.

Our sample showed males to have a longer clavicle (15.6 cm) than females (14.3 cm). Similarly, sternal diameter, midshaft diameter, and acromial diameter were all greater in males of our sample, indicating that males have more robust clavicles than females. Identical results were reported in Andermahr et al.'s study [16]. The male population is on average taller, which can explain the longer clavicle, increased radius of curvature, and greater diameter of that population compared with females. The results of relationship between height and clavicular anatomical parameters were shown in Table 3. All of the parameters were related to height except lateral curve depth (*S*6) and radius (*R*2). Accordingly, the difference in clavicle parameters may be solely related to height. Age, gender, and BMI do not influence the relationship between the height and clavicle parameters. As height increases, the depth and radius of medial curve of clavicle will increase as well. Oppositely, people with small stature have small radius of curvature of clavicle meaning the geometry of the bone is more curved. For the design of size distribution of precontoured clavicle bone plate, the curvature of bone plate should vary as the length of plate changes to achieve proper bone/plate fitness on the superior surface of the clavicle.

There are several limitations in this study. Firstly, we received only gender, age, height, and weight information in the original data. Generally, lifestyle, muscle action, and disease affect bony morphology. The correlation between these factors is still unknown. Secondly, we included only Chinese population as our subjects, and only a hundred subjects should not represent the whole Asian or Eastern population. More subjects should be enrolled for a more complete evaluation. Finally, several authors had found a difference in clavicle length between sides. Only right clavicles were studied in our sample. In a 699-case study, Terry found 61% of cases had longer left clavicles [18]. Martin and Saller also found the left clavicle tends to be longer than the right [19]. Also, data on the handedness in the samples were unavailable. Such information could have been useful for the left-right analysis.

## 5. Conclusion

Morphometric measurements of Chinese clavicles showed some differences in comparison to Caucasians'. These data may help to manufacture bone plates suitable for Chinese clavicles. Our results also show a significant correlation between the height and the medial clavicle curve. It should be noted that design of the precontoured clavicular bone plate may have to consider adapting the medial curvature to different length of bone plate to fit subject with different height.

## Figures and Tables

**Table 1:** Basic information of the subjects.

Parameters	All (*N* = 100)	Male (*N* = 50)	Female (*N* = 50)
Age	36.99 ± 9.28	34.84 ± 8.13	39.14 ± 9.83
Height (cm)	165.32 ± 7.73	171.14 ± 5.45	159.5 ± 4.69
Weight (kg)	65.92 ± 12.60	74.00 ± 11.01	57.84 ± 8.11

**Table 2:** Clavicle morphology-difference between male and female (mean ± SD).

Parameters (cm)	All (*N* = 100)	Male (*N* = 50)	Female (*N* = 50)	*P* value
Length (*S*1)	14.9 ± 1.3	15.6 ± 0.9	14.3 ± 1.3	<0.001^*∗*^
Sternal diameter (*S*2)	2.4 ± 0.3	2.5 ± 0.3	2.3 ± 0.3	<0.01^*∗*^
Middle third diameter (*S*4)	1.3 ± 0.2	1.4 ± 0.1	1.2 ± 0.1	<0.001^*∗*^
Acromial diameter (*S*3)	2.4 ± 0.4	2.6 ± 0.4	2.2 ± 0.3	<0.001^*∗*^
Medial curve depth (*S*5)	1.6 ± 0.3	1.7 ± 0.3	1.5 ± 0.3	<0.001^*∗*^
Medial curve radius (*R*1)	7.2 ± 1.5	7.5 ± 1.4	6.8 ± 1.6	<0.05^*∗*^
Lateral curve depth (*S*6)	1.2 ± 0.8	1.3 ± 1.1	1.0 ± 0.2	<0.05^*∗*^
Lateral curve radius (*R*2)	3.5 ± 1.7	3.4 ± 1.7	3.7 ± 1.8	0.431

**Table 3:** The relation between clavicular parameters and height.

Dependent variable	Estimate (SE)	*P* value
Length (*S*1)	0.84 (0.14)	<0.0001^*∗*^
Sternal diameter (*S*2)	0.13 (0.04)	0.002^*∗*^
Middle third diameter (*S*4)	0.16 (0.02)	<0.0001^*∗*^
Acromial diameter (*S*3)	0.23 (0.04)	<0.0001^*∗*^
Medial curve depth (*S*5)	0.12 (0.04)	0.004^*∗*^
Medial curve radius (*R*1)	0.47 (0.19)	0.018^*∗*^
Lateral curve depth (*S*6)	0.11 (0.10)	0.280
Lateral curve radius (*R*2)	0.03 (0.23)	0.879

**Table 4:** Review of the morphometry of the clavicle (in cm) in different studies.

Parameters (cm)	Our study (*N* = 100)	Andermahr et al. (*N* = 196)	Duprey et al. (*N* = 6)
Length (*S*1)	14.9 ± 1.3	15.1 ± 1.1	14.7 ± 0.9
Sternal diameter (*S*2)	2.4 ± 0.3	2.5 ± 0.4	2.6 ± 0.3
Middle third diameter (*S*4)	1.3 ± 0.2	1.2 ± 0.2	1.1 ± 0.3
Acromial diameter (*S*3)	2.4 ± 0.4	2.2 ± 0.4	2.7 ± 0.4
Medial curve depth (*S*5)	1.6 ± 0.3	1.7 ± 0.3	1.9 ± 0.4
Medial curve radius (*R*1)	7.2 ± 1.5	7.1 ± 1.3	6.2 ± 0.8
Lateral curve depth (*S*6)	1.2 ± 0.8	1.2 ± 0.3	1.3 ± 0.2
Lateral curve radius (*R*2)	3.5 ± 1.7	3.9 ± 1.4	2.8 ± 0.7
